# Artificial intelligence in (gastrointestinal) healthcare: patients’ and physicians’ perspectives

**DOI:** 10.1038/s41598-022-20958-2

**Published:** 2022-10-06

**Authors:** Quirine E. W. van der Zander, Mirjam C. M. van der Ende - van Loon, Janneke M. M. Janssen, Bjorn Winkens, Fons van der Sommen, Ad. A. M. Masclee, Erik J. Schoon

**Affiliations:** 1 Division of Gastroenterology and Hepatology, Maastricht University Medical Center, Maastricht, The Netherlands; 2 GROW, School for Oncology and Reproduction, Maastricht University, Maastricht, The Netherlands; 3 Division of Gastroenterology and Hepatology, Catharina Hospital Eindhoven, Eindhoven, The Netherlands; 4 Department of Methodology and Statistics, Maastricht University, Maastricht, The Netherlands; 5 CAPHRI, Care and Public Health Research Institute, Maastricht University, Maastricht, The Netherlands; 6 Department of Electrical Engineering, Eindhoven University of Technology, Eindhoven, The Netherlands

**Keywords:** Gastroenterology, Health care, Medical research

## Abstract

Artificial intelligence (AI) is entering into daily life and has the potential to play a significant role in healthcare. Aim was to investigate the perspectives (knowledge, experience, and opinion) on AI in healthcare among patients with gastrointestinal (GI) disorders, gastroenterologists, and GI-fellows. In this prospective questionnaire study 377 GI-patients, 35 gastroenterologists, and 45 GI-fellows participated. Of GI-patients, 62.5% reported to be familiar with AI and 25.0% of GI-physicians had work-related experience with AI. GI-patients preferred their physicians to use AI (mean 3.9) and GI-physicians were willing to use AI (mean 4.4, on 5-point Likert-scale). More GI-physicians believed in an increase in quality of care (81.3%) than GI-patients (64.9%, χ^2^(2) = 8.2, *p* = 0.017). GI-fellows expected AI implementation within 6.0 years, gastroenterologists within 4.2 years (t(76) =  − 2.6, *p* = 0.011), and GI-patients within 6.1 years (t(193) =  − 2.0, *p* = 0.047). GI-patients and GI-physicians agreed on the most important advantages of AI in healthcare: improving quality of care, time saving, and faster diagnostics and shorter waiting times. The most important disadvantage for GI-patients was the potential loss of personal contact, for GI-physicians this was insufficiently developed IT infrastructures. GI-patients and GI-physicians hold positive perspectives towards AI in healthcare. Patients were significantly more reserved compared to GI-fellows and GI-fellows were more reserved compared to gastroenterologists.

## Introduction

People living in western countries are facing artificial intelligence (AI) on a daily basis via facial recognition applications and speech processing tools. Recent developments in AI have led to the large-scale use of computer algorithms. Due to these successes, AI is starting to find practical applications in healthcare. AI can play a role in assisting physicians by providing (faster/more accurate) diagnoses, directing personalized treatment, making risk predictions, stratify diseases according to disease severity, and reducing medical errors^1,2^.

AI has great potential in imaging analysis. Examples within gastrointestinal (GI) endoscopy include detection and classification of colorectal lesions^3^, differentiation between superficial and deep invasive colorectal cancer^4^, disease severity scoring of inflammatory bowel diseases^5^, localizing blind spots during esophagogastroduodenoscopy^6^, and detecting Barrett’s neoplasia^7^. Some of these AI-systems diagnose diseases with expert-level accuracy or even outperform human experts^7–9^.

AI-based systems can also be used in personalized healthcare^10^. Labovitz et al. (2017) showed that AI is helpful in improving compliance to therapy^11^. Furthermore, AI systems do not get distracted, are not influenced by fatigue, and can perform certain tasks with greater consistency, speed, and reproducibility than physicians^2^. Therefore, AI can potentially lead to an optimized care trajectory, increasing healthcare efficiency and quality, and save healthcare costs^12^.

Despite the successes of AI in assisting in clinical tasks there is still some apprehension about the use of AI in healthcare by both patients and physicians. For smooth implementation, physicians need to have knowledge and willingness to use AI. Patients need to trust their physicians in using these techniques. AI product developers in healthcare, in turn, need to know the current bottlenecks and apprehensions in order to develop their products in such way that an optimal collaboration and joint performance between AI and physicians and between AI and patients is guaranteed. Since an intervention is only as successful as the target audience’s acceptance to the intervention, physicians and patients need to have or gain confidence in AI prior to optimal implementation in healthcare^13^. The primary aim of this study was to investigate the perspectives of GI-patients, gastroenterologists, and GI-fellows towards AI in healthcare.

## Methods

This non-interventional, prospective, questionnaire study was in accordance with the declaration of Helsinki and the General Data Protection Regulation. The Medical Ethical Review Committee of Maastricht UMC + (METC2020-2281) and Catharina Hospital Eindhoven (W20.017, February 2020) approved the study (ClinicalTrials.gov NCT05214625).

### Subjects

GI-patients who underwent an endoscopic procedure at Maastricht UMC + or Catharina Hospital Eindhoven between April 2020 and August 2021 and aged ≥ 18 years, were eligible for inclusion. Physicians were gastroenterologists and GI-fellows from multiple Dutch hospitals. Participants were only included if they had appropriate understanding of the Dutch language and were able to read, understand, and fill in the Dutch questionnaire. There were no exclusion criteria for participation. Each participant could participate in the study only once, without follow-up. All GI-patients and GI-physicians provided written informed consent prior to participation. No incentives were offered.

### Outcomes and questionnaires

The primary outcome was the perspective, defined as knowledge, experience, and opinion, of GI-patients, gastroenterologists, and GI-fellows on AI in healthcare and possible differences between their perspectives. Secondary outcomes included the willingness to implement AI in healthcare and important (dis)advantages of AI use. Secondary outcomes only investigated among GI-physicians included the willingness to use AI, the preferred domains for AI use in healthcare, the use of imaging enhancement techniques during endoscopy, and the availability of the mandatory infrastructure for AI implementation. Data were obtained using self-assessed, paper questionnaires collecting both quantitative and qualitative data. GI-patients and GI-physicians were provided with different questionnaires. To the best of our knowledge, no validated questionnaire for the objective of our study existed at the time of execution of this study. Therefore, questionnaires were developed according to the checklist for reporting of survey studies after reviewing literature (Supplementary Methods S1 and S2). Perspectives on AI and availability of the infrastructures were investigated using closed-ended (‘yes’, ‘no’, or ‘I don’t know’) and open questions. Responses concerning opinion and willingness were given on a 5-point Likert-scale, ranging from strongly disagree (1) to strongly agree (5). Questions regarding (dis)advantages of AI and domains in healthcare were multiple response questions in which a maximum of three answers could be chosen. In the questionnaire AI was explained briefly (Supplementary Methods S3). Questionnaires were handed out to patients during a visit at the outpatient clinic. GI-physicians completed the questionnaire during a yearly training day.

### Statistical analyses

Sample size calculations were performed using www.checkmarket.com/sample-size-calculator. To estimate a proportion (e.g. knowledge on AI) with a margin of error of 5% and a confidence level of 95%, 377 GI-patient and 209 GI-physician respondents were needed. All questionnaires were taken into account, including incomplete questionnaires. Baseline characteristics are presented as proportions (%) for categorical variables or as mean (standard deviation [SD]) for numerical variables. Multiple response questions were analyzed using descriptive statistics and reported as percentages of the total number of answers (%answers) and percentages of the GI-patients or GI-physicians that selected these answers (%GI-patients, %GI-physicians). For normally distributed data, differences between (sub)groups were analyzed using Chi-square test or Fisher’s exact test for categorical variables and independent sample t-test for numerical variables. The Mann–Whitney U test was used for non-normal distributions. Two-sided *p*-values ≤ 0.05 were considered statistically significant. Statistical analyses were performed with IBM SPSS Statistics (IBM Corp., Armonk, NY, USA).

## Results

### Study population

In total, 377 GI-patients participated of which 257 (68.2%) handed in a fully completed and 120 (31.8%) a partially completed questionnaire. The most prevalent indication for an endoscopic procedure was a colonoscopy because of the national screening program for colorectal cancer (61.5%, n = 232) (Table 1). The majority of GI-patients (94.1%, n = 351) used at least one electronic device in the past month. Computers and smartphones were used most. Devices were used for medical purposes by 44.5% (n = 157) of GI-patients (defined as users), while 55.5% (n = 196) never used a device for medical purposes (non-users). The purposes of medical device use are listed in Table 1. Of GI-patients, 62.5% (n = 228) reported to be familiar with AI. Patients (n = 258) reported associated words as ‘robot’ (31.0%, n = 80), ‘computer’ (23.6%, n = 61), and ‘digitalization’, ‘automation’, or ‘information technology’ (14.3%, n = 37). GI-patients with complete questionnaires had a significantly higher level of education, underwent significantly more often a colonoscopy because of screening, significantly more often were (medical) device users, and significantly more often were familiar with AI.

**Table 1:** Baseline characteristics for GI-patients.

	GI-patients N = 377
Gender, female n (%)	155 (41.1)
Age in years, mean (SD)	64.5 (20.8)
**Level of education, n (%) (N = 372)**	
Elementary education	35 (9.4)
Secondary education	211 (56.7)
Higher education	126 (33.9)
**Indication for endoscopic procedure, n (%)**	
CRC screening colonoscopy	232 (61.5)
Symptoms or surveillance*	145 (38.5)
**Device use, yes n (%) (N = 373)**	**351 (94**.**1)**
Computer or laptop	321 (86.1)
Smartphone	303 (81.2)
Smartwatch	65 (17.4)
Medical device use, yes n (%) (N = 353)	157 (44.5)
**Purpose of medical device use, yes n (%**^**) (N = 144)**	
Communication with physicians	26 (18.1)
Searching information	79 (54.9)
Tracking heartbeat and blood pressure	32 (22.2)
Tracking sport activities	16 (11.1)
Making appointments	5 (3.5)
Access to medical file	12 (8.3)
Monitor disease activity	8 (5.6)
Reminders for medication use	6 (4.2)
Other	11 (7.6)
Familiar with AI, yes n (%) (N = 365)	228 (62.5)

*Endoscopic procedures for symptoms or because of surveillance were both gastroscopies and colonoscopies.

^Percentage of GI-patients using a medical device for this purpose.

*AI* artificial intelligence; *CRC* colorectal cancer; *GI* gastrointestinal; *SD* standard deviation.

In total, 35 gastroenterologists and 45 GI-fellows fully completed the questionnaire. The majority of gastroenterologists (82.9%, n = 29) used medical applications in their clinical work, in contrast to 57.8% (n = 26, χ^2^(1) = 5.8, *p* = 0.016) of GI-fellows (Table 2). Applications used by more than five GI-physicians are listed in Supplementary Table S3. Work-related experience with AI was reported by 37.1% (n = 13) of gastroenterologists and by 15.6% (n = 7) of GI-fellows. Personal exposure with AI was mainly research related (n = 6).

**Table 2:** Baseline characteristics for GI-physicians.

	Gastroenterologists N = 35	GI-fellows N = 45	*p* value
Gender, female n (%)	13 (37.1)	33 (73.3)	0.001
Age in years, mean (SD)	49.7 (7.6)	32.7 (2.9)	< 0.001
Year of education, n (%)*			
Year 2	–	1 (1.3)	–
Year 3	–	19 (42.2)	–
Year 4	–	10 (22.2)	–
Year 5	–	9 (20.0)	–
Year 6	–	6 (13.3)	–
Application use in clinical (GI) work, yes n (%)	29 (82.9)	26 (57.8)	0.016
Experience with AI in clinical (GI) work, yes n (%)	13 (37.1)	7 (15.6)	0.079

*No GI-fellows were in the first year of their education.

*App* mobile application; *GI* gastrointestinal; *SD* standard deviation.

### GI-patients’ perspectives

On a 5-point Likert-scale, GI-patients preferred their physicians to use AI (mean 3.9 [SD 1.0]) in their clinical work (Table 3). On average, GI-patients expected AI implementation in healthcare within 6.1 years (SD 4.6). The majority of GI-patients was not anxious for AI (68.8%, n = 238) and thought that implementation of AI in healthcare will increase the quality of care (64.9%, n = 231). Subgroup analyses showed that GI-patients reporting to be familiar with AI (62.5%, n = 228) had a significantly more positive perspective towards AI compared to GI-patients unfamiliar with AI. Their preference of AI use by their physicians was 4.0 (SD 1.0 vs 3.6 [SD 1.0], t(343) = -2.8, *p* = 0.005), they expected AI implementation within 5.6 years (SD 4.4 vs 7.7 [SD 5.5], t(116) = 3.0, *p* = 0.003), more believed in an increase in quality of care with AI (76.4% [n = 172] vs 45.0% [n = 58], χ^2^(2) = 35.8, *p* < 0.001), and only a few were anxious for AI (2.8% [n = 6] vs 8.1% [n = 10], χ^2^(2) = 27.5, *p* < 0.001) (Supplementary Table S4). Patients with fully completed questionnaires were also significantly more positive towards AI regarding AI use by their physicians, increase in quality of care, and anxiety compared to patients with partially completed questionnaires (Supplementary Table S4). The same accounted for male gender. Subgroup analysis for medical device use only showed a significantly earlier expectation of AI implementation for users compared to non-users. Higher level of education showed a positive trend towards AI compared to lower levels of education.

**Table 3:** Artificial intelligence in healthcare—GI-patients’ perspective.

	GI-patients N = 377
Willingness of AI use by physicians*, mean (SD) (N = 347)	3.9 (1.0)
**Years to implementation, mean (SD) [range] (N = 270)**	6.1 (4.6) [0–25]
5 years, n (%)	186 (68.9)
10 years, n (%)	64 (23.7)
15 years, n (%)	8 (3.0)
20 + years, n (%)	12 (4.4)
**Anxious for AI, n (%) (N = 346)**	
Yes	18 (5.2)
No	238 (68.8)
I don’t know	90 (26.0)
**Increase in quality of care with AI, n (%) (N = 356)**	
Yes	231 (64.9)
No	13 (3.7)
I don’t know	112 (31.5)

*On a 5-point Likert scale.

*AI* artificial intelligence; *GI* gastrointestinal; *SD* standard deviation.

Reported advantages of a virtual nurse, a technique performing tasks normally performed by nurses, were the availability at any time (GI-patients 50.0%, n = 177), the technique’s possibility to make appointments (GI-patients 49.4%, n = 175), and to control and monitor disease activity (GI-patients 35.0%, n = 124) (Supplementary Table S5). GI-patients preferred mobile applications as digital communication tool with their healthcare professionals (GI-patients 47.5%, n = 168), followed by text massages (GI-patients 26.6%, n = 94), and websites (GI-patients 26.0%, n = 92) (Supplementary Table S6).

### GI-physicians’ perspectives

GI-physicians expected their work to change by AI (gastroenterologists mean 4.8 [SD 0.4] vs GI-fellows mean 4.3 [SD 0.7], t(73) = 3.9, *p* < 0.001, on a 5-point Likert-scale) (Table 4). Gastroenterologists expected AI implementation in healthcare within 4.2 years (SD 2.7), while GI-fellows expected this within 6.0 years (SD 3.0, t(76) = -2.6, *p* = 0.011). GI-physicians were willing to use AI for their patients (mean 4.4 [SD 0.7]). The majority of GI-physicians believed that the implementation of AI in healthcare will increase the quality of care (81.3%, n = 65).

**Table 4:** Artificial intelligence in healthcare—GI-physicians’ perspective.

	GI-physicians N = 80	Gastro-enterologists N = 35	GI-fellows N = 45	*p* value^
Expectation of work changes by AI*, mean (SD)	4.5 (0.7)	4.8 (0.4)	4.3 (0.7)	< 0.001
**Years to implementation, mean (SD) [range]**	5.2 (3.0)	4.2 (2.7)	6.0 (3.0)	0.011
5 years, n (%)	61 (78.2)	29 (85.3)	32 (72.7)	–
10 years, n (%)	15 (19.2)	5 (14.7)	10 (22.7)	–
15 years, n (%)	2 (2.6)	0 (0.0)	2 (4.5)	–
20 + years, n (%)	0 (0.0)	0 (0.0)	0 (0.0)	–
Willingness to use AI as physician*, mean (SD)	4.4 (0.7)	4.6 (0.7)	4.3 (0.7)	0.014
Willingness for physicians to use AI as patient*, mean (SD)	4.1 (0.8)	4.2 (0.8)	4.0 (0.9)	0.243
**Increase in quality of care with AI, n (%)**				0.433
Yes	65 (81.3)	29 (82.9)	36 (80.0)	–
No	1 (1.3)	1 (2.9)	0 (0.0)	–
I don’t know	14 (17.5)	5 (14.3)	9 (20.0)	–

*On a 5-point Likert scale.

^*p* value reported for differences between gastroenterologists and GE fellows.

*AI* artificial intelligence; *GI* gastrointestinal; *SD* standard deviation.

Subgroup analyses among GI-physicians showed that more application users had a positive perspective towards AI than non-users. Their expectation of work changes by AI was 4.6 (SD 0.6) compared to 4.2 (SD 0.7) for non-users (t(78) = -2.3, *p* = 0.022). They expected earlier AI implementation (4.7 years [SD 2.4] vs 6.4 years [SD 3.8], t(32) = 2.0, *p* = 0.052), were more willing to use AI as physicians (mean 4.5 [SD 0.7] vs mean 4.2 [SD 0.7], t(78) = -1.7, *p* = 0.093), and more believed in an increase in quality of care with AI (85.5% [n = 47] vs 72.0% [n = 18], χ^2^(2) = 3.1, *p* = 0.209).

GI-physicians expect the most benefits of AI in the domain of diagnostics: diagnostics within endoscopy (72.5%, n = 58), diagnostics within radiology (61.3%, n = 49), and diagnostics within histopathology (45.0%, n = 36) (Table 5).

**Table 5:** Fields of application of AI in healthcare and domains within gastroenterology and hepatology.

	GI-physicians	
	n (% of physicians) N = 80	n (% of answers) N = 234*
Diagnostics—endoscopy	58 (72.5)	58 (24.8)
Diagnostics—radiology	49 (61.3)	49 (20.9)
Diagnostics—histopathology	36 (45.0)	36 (15.4)
Identify risk profiles	26 (32.5)	26 (11.1)
Telemonitoring	18 (22.5)	18 (7.7)
Education about diseases and patient self-management	13 (16.3)	13 (5.6)
Robot assisted treatment	12 (15.0)	12 (5.1)
(Personalized) treatment	12 (15.0)	12 (5.1)
Communication (virtual nurse)	10 (12.5)	10 (4.3)

*Multiple response questions.

*GI* gastrointestinal.

To investigate whether the infrastructure of GI-endoscopy in Dutch hospitals is ready for AI implementation, GI-physicians reported the ability to save endoscopic images and videos within their hospitals. In total, 85.0% (n = 68) of the GI-physicians had the ability to save endoscopic images in high definition quality and 71.3% (n = 57) for high definition videos. In addition, 92.5% (n = 74) could save those images in the electronic patient file (Table 6). The mean number of images taken during a colonoscopy and gastroscopy were similar for gastroenterologists and GI-fellows. Imaging enhancement techniques such as narrow band imaging, use specific wavelengths of light in order to optimize the visualization of vessels and mucosal patterns. The standard use of these imaging enhancement techniques was significantly lower among GI-fellows (48.9%, n = 22) compared to gastroenterologists (80.0% [n = 28], χ^2^(2) = 9.8, *p* = 0.007).

**Table 6:** Imaging during endoscopy.

	GI-physicians N = 80	Gastroenterologists N = 35	GI-fellows N = 45	*p* value
Ability to save HD images, yes n (%)*	68 (85.0)	–	–	–
Ability to save HD videos, yes n (%)*	57 (71.3)	–	–	–
Ability to save HD images in electronic patient file, yes n (%)*	74 (92.5)	–	–	–
Number of images taken per colonoscopy, mean (SD)	–	10.0 (4.8)	8.6 (4.1)	0.187
Number of images taken per gastroscopy, mean (SD)	–	7.3 (2.6)	7.6 (2.7)	0.695
Use of imaging enhancement techniques, yes n (%)	–	28 (80.0)	22 (48.9)	0.007

*Gastroenterologists and GI-fellows were working in the same hospitals. Therefore, only numbers for the total group (GI-physicians) are provided.

*GI* gastrointestinal; *HD* high definition; *SD* standard deviation.

### Comparing GI-patients and GI-physicians

GI-patients and GI-physicians both believed in a quality of care increase with AI, but significantly more GI-physicians were convinced (81.3%, n = 65) than GI-patients (64.9% [n = 231], χ^2^(2) = 8.2, *p* = 0.017). The expectation of GI-fellows was that AI will have a place in healthcare within 6.0 years (SD 3.0), whereas gastroenterologists expected this within 4.2 years (SD 2.7, t(76) = -2.6, *p* = 0.011, compared to GI-fellow) and GI-patients within 6.1 years (SD 4.6 vs 5.2 years [SD 3.0], t(193) = -2.0, *p* = 0.047, compared to GI-physicians). GI-patients and GI-physicians agreed on the most important advantages of AI in healthcare: improving quality of care (GI-patients 66.1% [n = 228] vs GI-physicians 90.0% [n = 72]), time saving (GI-patients 38.0% [n = 131] vs GI-physicians 55.0% [n = 44]), and faster diagnostics and shorter waiting times (GI-patients 71.3% [n = 246] vs GI-physicians 51.3% [n = 41]) (Table 7). The most important disadvantage for GI-patients was the potential loss of personal contact with healthcare professionals (66.4%, n = 227), where this was insufficiently developed information technology infrastructures for GI-physicians (56.3%, n = 45) (Table 8). For both GI-patients and GI-physicians this was followed by the lack of (technical) knowledge by physicians (GI-patients 27.8% [n = 95] vs GI-physicians 50.0% [n = 40]) and uncertainty about laws and regulations (responsibility) (GI-patients 48.5% [n = 166] vs GI-physicians 35.0% [n = 28]). A difference between gastroenterologists and GI-fellows was seen in the concern for the loss of skills by AI. None of the gastroenterologists reported this as a disadvantage, while it was reported by 42.2% (n = 19) of GI-fellows (Supplementary Table S7). A smaller difference in concerns between gastroenterologists and GI-fellows was seen for the loss of employment (gastroenterologists 0.0% [n = 0] vs GI-fellows 6.7% [n = 3]) and lack of human supervision (gastroenterologists 20.0% [n = 7] vs GI-fellows 28.9% [n = 13]).

**Table 7:** Advantages of artificial intelligence in healthcare—GI-patients’ and GI-physicians’ perspectives.

Advantages of AI	GI-patients			GI-physicians		
	n N = 345	% of patients N = 345	% of answers N = 1004*	n N = 80	% of physicians N = 80	% of answers N = 237*
Improving quality of care	228	66.1	22.7	72	90.0	30.4
Personalized care	54	15.7	5.4	22	27.5	9.3
Time saving (for the physicians)	131	38.0	13.0	44	55.0	18.6
Faster diagnostics and shorter waiting times (for the patient)	246	71.3	24.5	41	51.3	17.3
Solutions for complex care tasks	74	21.4	7.4	17	21.3	7.2
Availability at any time (24/7)	85	24.6	8.5	5	6.3	2.1
Remote communication	67	19.4	6.7	12	15.0	5.1
Education about diseases and health for the patient^	21	6.1	2.1	–	–	–
Education about diseases and health for physicians	27	7.8	2.7	8	10.0	3.4
Costs	62	18.0	6.2	13	16.3	5.5
No benefits	6	1.7	0.6	1	1.3	0.4
Other advantagesǂ	3	0.9	0.3	2	2.5	0.8

*Multiple response questions.

^Answer options not given to physicians.

ǂFor ‘other advantages’ patients reported continuity in treatment (n = 1), independent of humans (n = 1), and research (n = 1). Gastroenterologists reported a different healthcare perspective for patients (n = 1) and more control for physicians (n = 1).

AI: artificial intelligence; GI: gastrointestinal; IT: information technology.

**Table 8:** Disadvantages of artificial intelligence in healthcare—GI-patients’ and GI-physicians’ perspectives.

Disadvantages of AI	GI-patients			GI-physicians		
	n N = 342	% of patients N = 342	% of answers N = 861*	n N = 80	% of physicians N = 80	% of answers N = 214*
Loss of personal contact with physicians^	227	66.4	26.4	–	–	–
Fear that your physician is using the technique incorrectly^	57	16.7	6.6	–	–	–
Fear that you as a patient are using the technique incorrectly^	47	13.7	5.5	–	–	–
Lack of (technical) knowledge by physicians	95	27.8	11.0	40	50.0	18.7
Insufficiently developed IT infrastructure	78	22.8	9.1	45	56.3	21.0
Uncertainty about laws and regulations (responsibility)	166	48.5	19.3	28	35.0	13.1
Insufficient privacy protection	81	23.7	9.4	12	15.0	5.6
Insufficient support from hospital administration	10	2.9	1.2	10	12.5	4.7
Problems with health insurance reimbursement	39	11.4	4.5	8	10.0	3.7
Costs	23	6.7	2.7	20	25.0	9.3
No disadvantages	25	7.3	2.9	8	10.0	3.7
Other disadvantagesǂ	13	3.8	1.5	1	1.3	0.5
Loss of employmentф	–	–	–	3	3.8	1.4
Loss of skills^ф^	–	–	–	19	23.8	8.9
Lack of human supervision^ф^	–	–	–	20	25.0	9.3

*Multiple response questions.

^Answer options not given to physicians.

ǂFor ‘other disadvantages’ patients reported loss of expertise by the physicians (n = 5), unseen misdiagnosis (n = 3), cuts in healthcare (n = 3), loss of employment for physicians (n = 2). One gastroenterologist reported a loss of the human dimension (n = 1).

ФAnswer options not given to GI-patients.

## Discussion

This study compared the perspectives of GI-patients, gastroenterologists, and GI-fellows on artificial intelligence in healthcare. We showed that there is a general positive perspective towards AI and AI implementation in healthcare, but GI-patients were more reserved compared to GI-fellows and GI-fellows in their turn were more reserved compared to gastroenterologists.

AI-research has focused on studies investigating accuracy of AI-based systems, while there is a gap in knowledge on patients’ and physicians’ perspectives towards AI. Successful implementation of AI into routine clinical practice depends not only on technical challenges, but also on the public’s trust and acceptance of AI^14^. Trust in AI is determined by the way people interact with the technology and dependent on the ease of use, reliability, transparency, explainability, security and privacy protection, and communication on the use of AI systems^13^.

Here, GI-patients preferred their physicians to use AI (mean 3.9 on a 5-point Likert-scale) and GI-physicians were willing to use AI for their patients (mean 4.4). This positive attitude is largely consistent with literature^15–18^, although concerns were raised by Yakar et al. (2022) who observed distrust towards AI in medicine among the Dutch general population^19^. In the current study, gastroenterologists were significantly more progressive towards AI than GI-fellows. Gastroenterologists had higher expectations of their work to change by AI and believed in a significant faster implementation of AI compared to GI-fellows. These results are interesting and somewhat controversial since GI-fellows are from a younger generation raised with digitalisation compared to gastroenterologists. A possible explanation may be found in the reporting of deskilling, employability, and negative career impacts by GI-fellows, while gastroenterologists did not report these concerns. Literature also shows limited impact of those specific issues^18,20^. Furthermore, we might speculate that gastroenterologists oversee their own shortcomings, the field, and its impossibilities better than GI-fellows. Partly supported by the routine use of imaging enhancement techniques by gastroenterologists, but much less by GI-fellows.

In line with literature, the majority of GI-patients (68.9%) and GI-physicians (78.2%) expected implementation of AI in healthcare within five years^17,18^. GI-patients (64.9%) and GI-physicians (81.3%) believed that AI will improve quality of care, again comparable with literature^21^. Human interaction in addition to AI use was considered critical for the experience of high-quality care^22^. The importance of human interactions is further supported by evidence showing that patients’ compliance was higher for physicians and for physicians using AI compared to an AI-system alone^8^. This so called augmented intelligence emphasizes that AI enhances or assists human intelligence rather than replacing it, expressing the importance of symbiosis between humans and AI^16,23,24^.

Medical device use among patients was low compared to literature^15,23^ and did not show a positive trend towards AI for users compared to non-users. In contrast, perspectives of GI-patients familiar with AI were significantly more positive compared to those unfamiliar with AI. Familiarity led to a higher willingness of GI-patients for their physicians to use AI, an earlier expected implementation of AI, and more GI-patients believed in an increase in quality of care compared to GI-patients unfamiliar with AI. Familiarity was self-reported and as high as 62.5%, which is comparable to literature^20,25,26^. However, this means that still one third of patients was unfamiliar with artificial intelligence, leaving room for better dissemination of information. It was not investigated to what extend GI-patients were familiar with AI, while AI acceptance was found to be higher in patients who assigned a higher rating to their AI knowledge^27^. Castagno et al. (2020) showed that 87% of healthcare staff did not know the difference between machine learning and deep learning^20^. The fast evolutions and developments in AI may result in an overflow of information, unmanageable for patients and physicians. This may paradoxically discourage further developments and implementation, emphasizing the importance of education and training^14,17^.

Acceptance of AI is also driven by patients’ and physicians’ understanding of potential (dis)advantages^13^. Hence, in this study the most frequently mentioned advantages of AI in healthcare were improved quality of care and time saving for both patients and physicians. Other perceived advantages are reducing risks of medical errors, more time available for physician–patient interaction, standardization in the interpretation of results, more objective diagnosis, gain in efficiency, and reduced costs^17,23,28^. Important disadvantages of AI were insufficiently developed information technology infrastructures, potential loss of personal contact, lack of (technical) knowledge by physicians, and uncertainty about laws and regulations. Other perceived disadvantages are overdependence on AI, increased procedural time, privacy protection, lack of (non-)verbal communication, and increased costs^12,15,16,20,23,25,28,29^.

Current literature is inconclusive about the effects of AI on workload. AI use is believed to save time, time that physicians could invest in personal contact with their patients, improving the physician–patient relationship^16,25^. In contrast, others reported a distortion of the physician–patient relationship as a concern of AI^20,30^. Remarkably, time for physician–patient interaction, procedural time, and costs are both perceived advantages and disadvantages, highlighting the importance of clear information, education, and studies investigating these outcomes.

Agreement existed on the fields of application of AI. Diagnostics within endoscopy, radiology, and histopathology were reported most promising by GI-physicians. Previous studies among gastroenterologists showed high interest for AI-assistance in colorectal polyp detection and in capsule endoscopy^29,30^. In contrast to the interest of GI-physicians in AI in diagnostic processes, patients preferred physician decision makers over AI decision makers, resulting in lower levels of trust when decisions were made by AI rather than by humans^24^. In addition, patients’ expressed a significantly higher confidence in AI-assisted interpretation than in AI-assisted management^15^.

An important requirement for implementation of AI in clinical practice is the technical infrastructure to be aligned with AI needs. Servers, data storage capacity, and (endoscopic) equipment need to meet these demands. Routine use of high definition endoscopes and digital imaging enhancement techniques are recommended by the European Society of Gastrointestinal Endoscopy^31^. Gastroenterologists in this study routinely used imaging enhancement techniques (80.0%) compared to less than half of GI-fellows (48.9%). One reason for GI-fellows not routinely using these imaging enhancement techniques might be the lack of experience. Although the use of these techniques is in line with a survey among US gastroenterologists^29^, this may hamper the added value of AI since most endoscopic AI-systems are built on using these imaging techniques.

The results of the current study should be considered in light of potential limitations. Unfortunately, the sample size for GI-physicians was not reached leading to a larger margin of error. In the Netherlands, there are around 800 practicing GI-physicians. Since we only recruited GI-physicians during one single Dutch training day, including 209 GI-physicians was not feasible using this approach. However, we do consider our sample of 80 GI-physicians representative. Due to COVID-19 restrictions, inclusions were temporary discontinued. Therefore, the total inclusion period for GI-patients was ten months. Selection bias may have occurred as responders more likely held strong opinions (both positive and negative) towards AI or were either more or either less informed about AI than non-responders. Response bias cannot be excluded as participants may have given assumed desirable answers, although they were explicitly asked not to do so. The order of response options of multiple response questions were not randomized in the questionnaires. This may have caused bias due to the primacy and recency effects, the tendency to better remember information or response options that are presented first or last, respectively^32^. Furthermore, the framing effect (bias caused by the manner in which questions are presented by using positive or negative words) may have influenced patients’ responses^33^. We did not investigate how well informed respondents were on AI or if they understood or were aware of potential shortcomings of AI, while insufficient or incorrect information could have biased the answers. We included GI-patients and GI-physicians. Therefore, these results may not be directly generalizable to other patient groups or medical specialties. Answers were self-reported and the questionnaires were not validated.

In summary, both GI-patients and GI-physicians hold positive perspectives towards AI and AI implementation in healthcare. GI-patients are more reserved compared to GI-fellows and GI-fellows are more reserved compared to gastroenterologists. One third of patients was unfamiliar with AI. AI will only have a beneficial role in healthcare if patients and physicians are knowledgeable and supportive towards AI. Therefore, AI developments should be conducted in a patient and physician-centric manner. Misconceptions and perceived (dis)advantages should be conquered by better disseminating information in layman’s terms and by educating physicians and patients.

## Supplementary Information

Supplementary Information.

## Data Availability

The data that support the findings of this study are available from the corresponding author upon reasonable request. This data includes deidentified participant data. Additional documents that will be made available are the study protocol, the statistical analysis plan, the questionnaires, and the informed consent forms. Data will be available following publication with no end date. Requests should be methodologically sound proposals with the purpose to achieve aims in the approved proposal. Data requestors will need to sign a data access agreement after approval of a proposal.
