# Supplementary material for: Artificial intelligence in (gastrointestinal) healthcare: patients’ and physicians’ perspectives
Source: Sci Rep. 2022 Oct 6;12:16779. doi: 10.1038/s41598-022-20958-2 (PMC9537305; doi:10.1038/s41598-022-20958-2)
Supplement: Supplementary file 1 — Supplementary Information. [file 41598_2022_20958_MOESM1_ESM.pdf]

Supplementary Information

Artificial intelligence in (gastrointestinal) healthcare – Patients' and physicians' perspectives

Quirine EW van der Zander[1,2], Mirjam CM van der Ende - van Loon[3], Janneke MM Janssen[2], Bjorn Winkens[4,5], Fons van der Sommen[6], Ad AM Masclee[1], Erik J Schoon[2,3]

Table of contents

Supplementary Methods S1. Questionnaire on artificial intelligence in (gastrointestinal) healthcare for patients with gastrointestinal disorders. 2

Supplementary Methods S2. Questionnaire on artificial intelligence in (gastrointestinal) healthcare for gastroenterologists and fellows within Gastroenterology and Hepatology. 6

Supplementary Methods S3. Patient information form – Questionnaire on artificial intelligence in (gastrointestinal) healthcare for patients with gastrointestinal disorders. 10

Supplementary Table S3. Medical applications (apps) used by more than five GI-physicians. 11

Supplementary Table S4. Subgroup analyses for artificial intelligence in healthcare – GI-patients' perspective. 12

Supplementary Table S5. Advantages or tasks of a virtual nurse – GI-patient's perspective. 13

Supplementary Table S6. Preferences for a digital communication tool – GI-patients' perspective. 14

Supplementary Table S7. Advantages and disadvantages of artificial intelligence in healthcare – Gastroenterologists' and GI-fellows' perspectives. 15

Supplementary Methods S1. Questionnaire on artificial intelligence in (gastrointestinal) healthcare for patients with gastrointestinal disorders.

We kindly request you to answer the general questions below. Please read each question carefully.

1. What is your gender?
   - ❑ Male
   - ❑ Female
   - ❑ Other, ……………………………

2. What is your year of birth?

   …………………………………

3. What is the highest level of education you completed?
   - ❑ Primary education
   - ❑ High school
   - ❑ Secondary vocational education
   - ❑ Higher vocational education
   - ❑ University (of applied sciences)

4. Did you use any of the following devices in the past month?

- ❑ Yes, please specify:
  - ❑ Computer or laptop
  - ❑ Smartphone
  - ❑ Smartwatch
- ❑ No

5. If you answered question 4 with 'yes':
   Do you use this device(s) for medical purposes?
   For example, searching for disease-related information, measuring your heart rate, tracking medication intake, telemedicine (remote patient monitoring), or communication with your physician.

- ❑ Yes, namely because of:
  - ❑ ....
- ❑ No
- ❑ Not applicable (question 4 answered with 'no')

6. What is your experience with the department of gastroenterology and hepatology?
   - ❑ I am <u>currently</u> being treated by a gastroenterologist (including control appointments)
   - ❑ I have been treated by a gastroenterologist <u>in the past,</u> at the moment I am not receiving any treatment
   - ❑ I have undergone an endoscopic examination (gastroscopy or colonoscopy) or I am undergoing this soon
   - ❑ Other, …………………………………

7. Were you familiar with the term or concept of *artificial intelligence* before reading this questionnaire?
   - ❑ Yes
   - ❑ No

8. What word comes to mind when you think of artificial intelligence?
   Write down one word.

   …………………………………………………………………………………….

Artificial Intelligence

The following questions are specifically about artificial intelligence.

9. As patient, I would want my (gastroenterology) physician to use artificial intelligence

   Strongly disagree1 ☹ 2 3 4 5 Strongly agree ☺

10. Within how many years do you expect the work of physicians to change by artificial intelligence?
    …………………………………………………………………………………….

11. Do you think the quality of care will improve by the use of artificial intelligence?
    - ❑ Yes
    - ❑ No
    - ❑ I don't know

12. What are the 3 most important advantages of artificial intelligence for you?
    Tick a maximum of 3 answers:
    - ❑ Improve the quality of care
    - ❑ Personalized healthcare
    - ❑ Time savings (for physicians)
    - ❑ Quicker diagnoses and shorter waiting times (for patients)
    - ❑ Solutions for complex healthcare tasks
    - ❑ Availability 24/7
    - ❑ (Remote) communication
    - ❑ Education about diseases and health for patients
    - ❑ Education about diseases and health for physicians
    - ❑ Cost reduction
    - ❑ No advantages
    - ❑ Other, ……………………………………

13. What are the 3 most important disadvantages for you to use artificial intelligence?
Tick a maximum of 3 answers:

- ❑ Lack of (technical) expertise by the physician
- ❑ Insufficiently developed IT-infrastructure (information technology)
- ❑ Insecurity about legislation and regulations concerning responsibility in making medical decisions by artificial intelligence
- ❑ Insufficient protection of privacy
- ❑ Insufficient support of hospital management
- ❑ Problems concerning health insurance by the health insurer
- ❑ Loss of personal contact with the doctor (technique replaces physicians)
- ❑ Fear that your doctor is using the technique incorrectly
- ❑ Fear that you as a patient are using the technique incorrectly
- ❑ Costs
- ❑ No disadvantages
- ❑ Other, …………………………………

14. Are you anxious about artificial intelligence?
- ❑ Yes
- ❑ No
- ❑ I don't know

15. What are the 3 most important advantages or tasks a virtual nurse can offer to you as a patient?
A virtual nurse is a technique that carries out tasks that are normally carried out by a nurse.
Tick a maximum of 3 answers:

- ❑ Scheduling appointments
- ❑ Receiving results
- ❑ Available at any moment (24/7)
- ❑ Control and monitor disease activity
- ❑ Therapy compliance (adherence to advices and instructions in the right way)
- ❑ Remote communication (communicate at home instead of in the hospital)
- ❑ Providing information about a disease and treatment to a patient
- ❑ More time for an appointment with the physician
- ❑ Cost reduction
- ❑ No advantages
- ❑ Other, ……………………………

16. How would you prefer to communicate with a virtual nurse?
- ❑ Mobile application (app)
- ❑ Website
- ❑ Message service (for example by short message service [SMS] or Whatsapp)
- ❑ Virtual voice (for example Siri or Google Assistant)
- ❑ Robot
- ❑ I don't know
- ❑ Other, ……………………………

17. Remarks

If you have any questions or remarks in response to this questionnaire, please write them down below:

…………………………………………………………………………………….
…………………………………………………………………………………….
…………………………………………………………………………………….
…………………………………………………………………………………….

You reached the end of the questionnaire.
We request you to hand in the questionnaire at the registration desk.

Thank you for your cooperation.

Supplementary Methods S2. Questionnaire on artificial intelligence in (gastrointestinal) healthcare for gastroenterologists and fellows within Gastroenterology and Hepatology.

We kindly request you to answer the general questions below. Please read each question carefully.

1. What is your gender?
   - ❑ Male
   - ❑ Female
   - ❑ Other, ……………………………………

2. What is your year of birth?

   ……………………………………

3. What is your function?
   - ❑ Gastroenterologist
   - ❑ Fellow within Gastroenterology and Hepatology
   - ❑ Other, ……………………………………

4. If you are a fellow within Gastroenterology and Hepatology, in which year of your education are you?
   - ❑ Year 1
   - ❑ Year 2
   - ❑ Year 3
   - ❑ Year 4
   - ❑ Year 5
   - ❑ Year 6
   - ❑ Not applicable

5. Where do you currently work?
   - ❑ University hospital
   - ❑ Non-university hospital
   - ❑ Private center
   - ❑ Other, ……………………………………

Artificial Intelligence

The following questions are about artificial intelligence.

6. As a gastroenterologist or GE fellow, I expect my work to change by the use of technologies based on artificial intelligence.

   Strongly disagree1 2 3 4 5 Strongly agree

   ☹ ☺

7. If you expect your work to change, within how many years do you expect this to happen?
Write down the number of years.

…………………………………………………………………………………….

8. As a gastroenterologist or GE fellow, I am willing to work with technologies based on artificial intelligence.

Strongly disagree1 2 3 4 5 Strongly agree
☹ ☺

9. What are the 3 most important <u>disadvantages</u> to the use of artificial intelligence for you?
Tick a maximum of 3 answers:

❑ Lack of (technical) expertise
❑ Legislation and regulations (medical ethical barriers) and responsibility
❑ Privacy
❑ IT-infrastructure
❑ Hospital management
❑ Health insurance
❑ Loss of working opportunities
❑ Loss of skills
❑ Costs
❑ Lack of human supervision
❑ No barriers
❑ Other, …………………………………

10. What are the 3 most important <u>advantages</u> to the use of artificial intelligence for you?
Tick a maximum of 3 answers:

❑ Improving the quality of care
❑ Personalized healthcare
❑ Time savings (for physicians)
❑ Quicker diagnoses and shorter waiting times (for patients)
❑ Solutions for complex healthcare tasks
❑ Availability 24/7
❑ (Remote) communication
❑ Education and teaching
❑ Cost reduction
❑ No advantages
❑ Other, …………………………………

11. Do you think the quality of care will improve by using technologies based on artificial intelligence?

❑ Yes
❑ No
❑ I don't know

12. As a patient, I would like my (gastroenterology) physician to use artificial intelligence.

    Strongly disagree1 2 3 4 5 Strongly agree

13. In which 3 gastroenterology areas do you envision the most opportunities for artificial intelligence?
    Tick a maximum of 3 answers:
    - ❑ Diagnosis (radiology)
    - ❑ Diagnosis (endoscopy)
    - ❑ Diagnosis (histopathology)
    - ❑ (Personalized) treatment
    - ❑ Robot assisted treatment
    - ❑ Communication (virtual nurse)
    - ❑ Telemedicine (remote patient monitoring)
    - ❑ Identify risk profiles
    - ❑ Education concerning disease understanding and self-management
    - ❑ Other, …………………………………

14. Do you currently use medical applications (apps) for clinical work as a gastroenterologist or GE fellow?
    - ❑ Yes
    - ❑ No

15. If you answered 'yes' to the previous question, please indicate below which application(s) you are using:
    ………………………………………………………………………………….
    ………………………………………………………………………………….
    ………………………………………………………………………………….
    ………………………………………………………………………………….

16. Is your institution currently paying attention to artificial intelligence?
    - ❑ Yes
    - ❑ No
    - ❑ I don't know

17. If you answered 'yes' to the previous question, please indicate below in which way(s) this is done:
    ………………………………………………………………………………….
    ………………………………………………………………………………….
    ………………………………………………………………………………….
    ………………………………………………………………………………….

18. Can you save digital endoscopy <u>images</u> in HD (High-Definition) quality in your institution?
    - ❑ Yes
    - ❑ No
    - ❑ I don't know
    - ❑ Not applicable

19. Can you save digital endoscopy <u>videos</u> in HD (High-Definition) quality in your institution?
    - ❑ Yes
    - ❑ No
    - ❑ I don't know
    - ❑ Not applicable

20. Can you store digital endoscopy images in the electronic patient file (electronic medical record) in your institution?
    - ❑ Yes
    - ❑ No
    - ❑ I don't know
    - ❑ Not applicable

21. How many images do you take per colposcopy on average?

    ……………………………………

22. How many images do you take per gastroscopy on average?

    ……………………………………

23. Do you routinely use imaging enhancement techniques during endoscopies?
    - ❑ Yes
    - ❑ No
    - ❑ Not applicable

24. Remarks
    If you have any questions or remarks in response to this questionnaire, please write them down below:

    ………………………………………………………………………………….
    ………………………………………………………………………………….
    ………………………………………………………………………………….
    ………………………………………………………………………………….

You have reached the end of the questionnaire.
We request you to hand in the questionnaire at the registration desk.

Thank you for your cooperation.

Supplementary Methods S3. Patient information form – Questionnaire on artificial intelligence in (gastrointestinal) healthcare for patients with gastrointestinal disorders.

Dear Sir/Madam,

Artificial intelligence

What is artificial intelligence? And how can this technology contribute in healthcare?

Artificial intelligence is the intelligence demonstrated by machines. These computers sole problems by using algorithms and pattern recognition. In this way, these machines mimic cognitive functions of the human brain. Artificial intelligence is increasingly used in our daily lives. For example, in facial recognition on photos on your mobile phone or computer, for virtual assistants, personalized advertisements where you are shown advertisements based on your previous searches, social media platforms, and self-driving cars.

What is the purpose of this research?

Artificial intelligence can be used for applications in healthcare. Some of these applications of artificial intelligence are already in an advanced stage such as healthcare apps on your mobile phone. Other techniques and their applications are still in their infancy. For instance, having a computer analyze images from a CT or MRI scan.

In this scientific study, we investigate how you, as a patient, think about artificial intelligence and its application in (gastrointestinal) healthcare. We ask you to complete this questionnaire. We would like to know what your opinion is, there are no right or wrong answers.

If you consent to participation in this study, please read the patient information carefully, and complete the informed consent form and the questionnaire enclosed.

[The patient information form continues with medical and ethical explanations of rights and obligations according to Dutch guidelines.]

Supplementary Table S3. Medical applications (apps) used by more than five GI-physicians.

	n (%)<br>N = 80	Purpose of the application	Developer
MedCalc®	18 (22.5)	Medical calculators, scoring systems, and guidelines	Healthcare Technology, New York, USA
Pharmacotherapeutic compass®	11 (13.8)	Drug information and prescribing aid	Health care institute the Netherlands (ZIN), Diemen, the Netherlands
NLcolosurvRL®	7 (8.8)	Colonoscopy surveillance advise	EverywhereIM B.V., Amsterdam, the Netherlands
Up to Date®	5 (6.3)	Medical resource	UpToDate Inc., USA

GI: gastrointestinal.

Supplementary Table S4. Subgroup analyses for artificial intelligence in healthcare – GI-patients' perspective.

	n	AI use by physician, mean (SD)	p value	n	AI implementation in years, mean (SD)	p value	n	Increase in quality of care, yes (%)	p value	n	Anxious for AI, yes (%)	p value
**Familiar with AI**			0.005			0.003			<0.001			<0.001
Yes	224	4.0 (1.0)	..	192	5.6 (4.4)	..	225	172 (76.4)	..	217	6 (2.8)	..
No	121	3.6 (1.0)	..	77	7.7 (5.5)	..	129	58 (45.0)	..	124	10 (8.1)	..
**Questionnaire**			<0.001			0.934			<0.001			<0.001
Complete questionnaire	257	4.0 (1.0)	..	257	6.2 (4.8)	..	257	193 (75.1)	..	257	7 (2.7)	..
Incomplete questionnaire	90	3.5 (1.2)	..	13	6.3 (4.9)	..	99	38 (38.4)	..	90	11 (12.2)	..
**Medical device use**			0.117			0.011			0.099			0.065
Users	152	4.0 (1.0)	..	126	5.4 (4.4)	..	153	110 (71.9)	..	147	4 (2.7)	..
Non-users	179	3.8 (1.1)	..	134	6.9 (5.0)	..	184	112 (60.9)	..	181	13 (7.2)	..
**Level of education**									0.002			0.022
Elementary education	27	3.6 (1.0)	0.539*	13	6.9 (6.6)	0.783*	29	13 (44.8)	..	29	2 (6.9)	..
Secondary education	194	3.8 (1.1)	..	147	7.3 (5.2)	..	198	120 (60.6)	..	190	11 (5.8)	..
Higher education	123	4.1 (1.0)	0.042**	109	4.7 (3.5)	<0.271**	124	97 (78.2)	..	122	3 (2.5)	..
**Gender**			<0.001			0.244			0.001			0.001
Male	200	4.0 (0.9)	..	169	5.9 (4.7)	..	207	151 (72.9)	..	206	7 (3.4)	..
Female	147	3.6 (1.2)	..	101	6.6 (5.1)	..	148	80 (54.1)	..	139	11 (7.9)	..
**Colonoscopy indication**			0.520			0.522			0.008			0.196
National screening program	218	3.9 (1.0)	..	180	6.3 (5.1)	..	221	157 (71.0)	..	218	9 (4.1)	..
History of GI disease or surveillance	129	3.8 (1.0)	..	90	5.9 (4.3)	..	135	74 (54.8)	..	128	9 (7.0)	..

* Elementary education compared with secondary education.

** Elementary education compared with higher education.

AI: artificial intelligence; GI: gastrointestinal; SD: standard deviation.

Supplementary Table S5. Advantages or tasks of a virtual nurse – GI-patient's perspective.

	**GI-patients**		
	**n**<br>N = 354	**% of patients**<br>N = 354	**% of answers**<br>N = 984*
Availability at any time (24/7)	177	50.0	18.0
Make appointments	175	49.4	17.8
Control and monitor disease activity	124	35.0	12.6
Remote communication	114	32.3	11.6
Receiving results	97	27.4	9.9
More time for an appointment with physicians	96	27.1	9.8
Provide information about diseases & treatment to patients	93	26.3	9.5
Cost reduction	51	14.4	5.2
Therapy compliance	43	12.1	4.4
No benefits	14	4.0	1.4

*Multiple response questions.
GI: gastrointestinal.

Supplementary Table S6. Preferences for a digital communication tool – GI-patients' perspective.

	**GI-patients**		
	**n**<br>N = 354	**% of patients**<br>N = 354	**% of answers**<br>N = 444*
Mobile application (app)	168	47.5	37.8
Text message (e.g. SMS or Whatsapp^)	94	26.6	21.2
Website	92	26.0	20.7
Virtual voice (e.g. Siri[‡] or Alexa [ϕ])	15	4.2	3.4
Robot	11	3.1	2.5
I don't know	45	12.7	10.1
Other[¶]	19	5.4	4.3

*Multiple response questions. The total number of answers is higher than the total number of patients, since some patients by mistake ticked more than one answer option.
^Whatsapp®, Facebook Inc, Massachusetts, USA.
[‡]Siri®, Apple Inc, California USA.
[ϕ]Alexa®, Amazone.com, Inc, Seattle, USA.
[¶]For 'other' GI-patients reported not to be able or willing to use digital tools (n=11), to prefer video calls (n=4), telephone calls (n=3), or e-mails (n=1).
GI: gastrointestinal; SMS: short message service.

Supplementary Table S7. Advantages and disadvantages of artificial intelligence in healthcare – Gastroenterologists' and GI-fellows' perspectives.

	**Gastroenterologists**			**GI-fellows**		
**Advantages of AI**	**n**<br>N = 35	**% of gastro-enterologists**<br>N = 35	**% of answers**<br>N = 103*	**n**<br>N = 45	**% of GI-fellows**<br>N = 45	**% of answers**<br>N = 134*
Improving quality of care	31	88.6	30.1	41	91.1	30.6
Personalized care	11	31.4	10.7	11	24.4	8.2
Time saving (for the physicians)	16	45.7	15.5	28	62.2	20.9
Faster diagnostics and shorter waiting times (for the patient)	14	40.0	13.6	27	60.0	20.1
Solutions for complex care tasks	11	31.4	10.7	6	13.3	4.5
Availability at any time (24/7)	2	5.7	1.9	3	6.7	2.2
Remote communication	7	20.0	6.8	5	11.1	3.7
Education about diseases and health for physicians	4	11.4	3.9	4	8.9	3.0
Costs	4	11.4	3.9	9	20.0	6.7
No benefits	1	2.9	1.0	0	..	..
Other advantages^	2	5.7	1.9	0	..	..
**Disadvantages of AI**	**n**<br>N = 35	**% of patients**<br>N = 35	**% of answers**<br>N = 92*	**n**<br>N = 45	**% of physicians**<br>N = 45	**% of answers**<br>N = 122*
Lack of (technical) knowledge by physicians	17	48.6	18.5	23	51.1	18.9
Insufficiently developed IT infrastructure	25	71.4	27.2	20	44.4	16.4
Uncertainty about laws and regulations (responsibility)	10	28.6	10.9	18	40.0	14.8
Insufficient privacy protection	5	14.3	5.4	7	15.6	5.7
Insufficient support from hospital administration	4	11.4	4.3	6	13.3	4.9
Problems with health insurance reimbursement	6	17.1	6.5	2	4.4	1.6
Costs	14	40.0	15.2	6	13.3	4.9
No disadvantages	3	8.6	3.3	5	11.1	4.1
Loss of employment	0	..	..	3	6.7	2.5
Loss of skills	0	..	..	19	42.2	15.6
Lack of human supervision	7	20.0	7.6	13	28.9	10.7
Other disadvantages‡	1	2.9	1.1	0	..	..

*Multiple response questions.
^For 'other advantages' gastroenterologists reported a different healthcare perspective for patients (n=1) and more control for physicians (n=1).
‡For 'other disadvantages' one gastroenterologist reported a loss of the human dimension (n=1).
AI: artificial intelligence; GI: gastrointestinal; IT: information technology.
